# Deriving Three-Outcome Permutationally Invariant Bell Inequalities

**DOI:** 10.3390/e26100816

**Published:** 2024-09-25

**Authors:** Albert Aloy, Guillem Müller-Rigat, Jordi Tura, Matteo Fadel

**Affiliations:** 1 Institute for Quantum Optics and Quantum Information, Austrian Academy of Sciences, Boltzmanngasse 3, A-1090 Vienna, Austria; 2 Vienna Center for Quantum Science and Technology (VCQ), Faculty of Physics, University of Vienna, 1090 Vienna, Austria; 3 ICFO—Institut de Ciencies Fotoniques, The Barcelona Institute of Science and Technology, 08860 Castelldefels, Barcelona, Spain; 4 〈*aQa^L^*〉 Applied Quantum Algorithms Leiden, 2300 RA Leiden, The Netherlands; 5 Instituut-Lorentz, Universiteit Leiden, P.O. Box 9506, 2300 RA Leiden, The Netherlands; 6 Department of Physics, ETH Zürich, 8093 Zürich, Switzerland

**Keywords:** Bell inequalities, Bell nonlocality, Bell correlations, multipartite quantum correlations

## Abstract

We present strategies to derive Bell inequalities valid for systems composed of many three-level parties. This scenario is formalized by a Bell experiment with *N* observers, each of which performs one out of two possible three-outcome measurements on their share of the system. As the complexity of the set of classical correlations prohibits its full characterization in this multipartite scenario, we consider its projection to a lower-dimensional subspace spanned by permutationally invariant one- and two-body observables. This simplification allows us to formulate two complementary methods for detecting nonlocality in multipartite three-level systems, both having a complexity independent of *N*. Our work can have interesting applications in the detection of Bell correlations in paradigmatic spin-1 models, as well as in experiments with solid-state systems or atomic ensembles.

## 1. Introduction

Bell nonlocality is a form of quantum correlation that enables some tasks inaccessible by classical means, thus constituting a key resource for quantum technologies [1,2]. Revealing the presence of nonlocality in a system is therefore of great importance, for both fundamental reasons and practical applications.

Nonlocality is typically detected by the violation of a Bell inequality, which is a criterion satisfied by any possible form of correlations with a classical explanation, such as common agreements and shared randomness. Significant effort has been put into deriving useful and practical Bell inequalities, especially in the bipartite scenario [3,4]. On the contrary, Bell inequalities valid for a large number of parties are much less explored because characterizing the set of classical correlations becomes an intractable task even for a few parties. This can be the case already for N=2 parties, if the number of measurements or of possible outcomes is large [5]. Progress in the multipartite scenario has focused on particular classes of quantum states or Bell inequalities. Notable examples include [6,7,8] in the context of graph states [9] and GHZ states in particular [10,11], as well as genuinely entangled subspaces [12,13]; recent constructions on symmetric multipartite inequalities, e.g., in [14] that leverage the Gilbert algorithm [15] and in the context of spin chains [16,17,18] and bosonic qubits [19]; recursive constructions such as [20,21,22]; and insights from renormalization procedures [23,24,25].

Therefore, a strategy to simplify this problem, which has proven to be successful, is to trade complexity for expressivity [2]. This can be carried out by projecting the set of classical correlations onto a lower-dimensional subspace, for example, by considering translational [26,27,28] or permutational [29,30,31,32,33,34,35] symmetries. For the multipartite scenario with two measurements and two outcomes, this approach has allowed for the derivation of Bell inequalities that enable the study of the connection between nonlocality and phase transitions [36,37], metrology [38], as well as the experimental detection of Bell correlations in spin-1/2 atomic ensembles [39,40]. Scenarios with more measurements [30,33] or more outcomes [41,42], on the other hand, are much less explored.

Systems composed by spin-1 particles or, more generally, of qutrits, play an important role in nuclear physics [43,44,45,46], ultracold atomic ensembles [47] and in solid-state physics [48,49,50]. This has motivated the development of ideas to simulate qudit Hamiltonians using trapped ions, superconducting circuits and ultracold atoms. For this reason, it is natural to ask whether Bell nonlocality can be detected in these multipartite systems, a task which inevitably requires many-outcome Bell inequalities.

**Figure 1:**
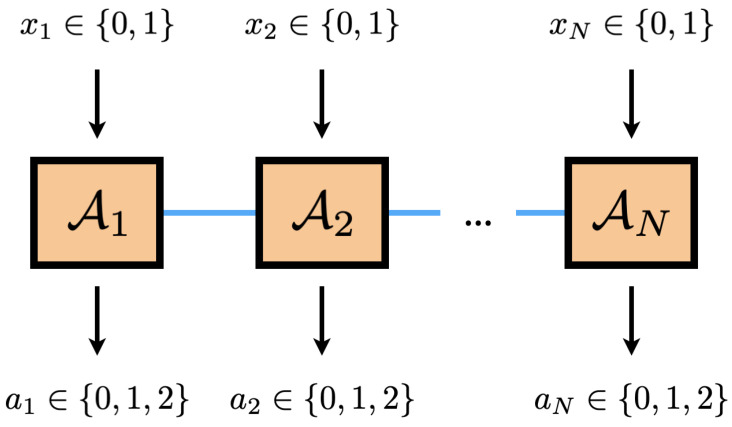
Schematic illustration of the (N,2,3) Bell scenario. Each party $A_{i}$ performs measurement $x_{i}$ and observes outcome $a_{i}$. After many repetitions, one estimates the conditional probability $p(a_{1}\ldots a_{N}|x_{1}\ldots x_{N})$ to check if it is compatible with a local hidden variable description. If this is not the case, Bell nonlocality is revealed.

In this work, we are interested in deriving Bell inequalities valid in a multipartite scenario with two measurements and three outcomes per party, see Figure 1. To make the problem tractable, we focus on finding inequalities involving permutationally invariant one- and two-body observables. Within this framework, we present two complementary strategies that allow for deriving new Bell inequalities. The first approach is based on the exact characterization of the (projected) set of classical correlations, and distilling classes of Bell inequalities that are valid for any *N*, while the second is based on a semidefinite program that approximates this set from the outside.

Our techniques find applications in the investigation of genuinely high-dimensional nonlocality in paradigmatic three-level Hamiltonians, such as three-orbital Lipkin–Meshkov–Glick models [43,46,51], as well as in the experimental detection of Bell correlations in spin-1 Bose–Einstein condensates [52,53,54].

## 2. Bell Scenario and the Local Polytope

### 2.1. Multipartite Bell Experiment

We consider a Bell experiment where *N* observers, labeled by i∈{1,…,N}, perform measurements on a physical system they share. Each observer can choose to perform one out of two possible local measurements $x_{i}\in \left[m\right]=\left\{0,1,\ldots ,m-1\right\}$, and each measurement will yield one of three possible outcomes $a_{i}\in \left[d\right]=\left\{0,1,\ldots ,d-1\right\}$. This is known as the (N,m,d) Bell scenario [1]. In the current work, we are interested in the (N,2,3) scenario, see Figure 1.

The full Bell experiment then takes place over several runs. At each run, a new copy of the physical system is distributed among the parties who will then choose a measurement to be implemented on their share and collect the resulting outcomes. Importantly, the measurement choice is independent from the state of the system, and the parties are not allowed to communicate while the experiment takes place.

After many runs, it is possible to estimate the conditional probability distribution p(a|x) for the observed statistics, which describes the probability of obtaining outcomes $a:=(a_{1},a_{2},\ldots ,a_{N})$ when measurements $x:=(x_{1},x_{2},\ldots ,x_{N})$ are chosen. This distribution satisfies p(a|x)≥0 as well as the normalization condition

(1) $$\sum_{a}p\left(a|x\right)=1\;\forall x\;.$$

Moreover, since the choice of a measurement input on party *i* cannot instantaneously signal information to the rest of the parties, the marginal probability distribution observed by any subset of the parties cannot depend on the measurement choices of the rest. This no-signaling (NS) principle implies the following constraint for the conditional probabilities

(2) ∑aip(a1,…,ai,…,aN|x1,…,xi,…,xN)AA=∑aip(a1,…,ai,…,aN|x1,…,xi′,…,xN),

for all *i* and $x_{i},x_{i}^{\prime }$. Therefore, *l*-body marginals $p(a_{k_{1}}\ldots a_{k_{l}}|x_{k_{1}}\ldots x_{k_{l}})$ are well defined on any subset $\left\{k_{1},\ldots ,k_{l}\right\}\subseteq \left\{1,\ldots ,N\right\}$.

Classical correlations imply that p(a|x) can be explained by pre-established agreements, i.e., by a local hidden variable model (LHVM), meaning that [3]

(3) $$p\left(a|x\right)\;\;\overset{LHVM}{=}\;\int p\left(\lambda \right)p\left(a_{1}|x_{1},\lambda \right)\ldots p\left(a_{N}|x_{N},\lambda \right)d\lambda \;,$$

where p(λ) is the probability of using agreement λ. If this is not the case, namely, if the observed statistics p(a|x) cannot be written as in Equation (3), we have to conclude the presence of Bell correlations in the system, i.e., nonlocality.

The set of p(a|x) for all a and x can be interpreted as a real vector in a probability space. Crucially, in this geometrical picture, the set of LHVM correlations is a polytope P, i.e., a bounded, closed, convex set with a finite number of extreme points, called vertices. P can be described by such a finite number of vertices or, equivalently, by the intersection of a finite number of half-spaces. These half-spaces are defined by linear inequalities that take the name of Bell inequalities. Concluding the presence of Bell nonlocality thus consists in deciding (non-)membership in the local polytope P. In the following sections, we propose strategies to tackle this membership problem for the (N,2,3) scenario.

### 2.2. Local Deterministic Strategies and Characterization of the Local Polytope

The vertices of the local polytope P correspond to a special class of LHVM, namely local deterministic strategies (LDSs) [55]. These satisfy

(4) $$\begin{array}{cc} \; & p\left(a|x\right)=p\left(a_{1}|x_{1}\right)\cdots p\left(a_{N}|x_{N}\right)\;\left(\mathrm{local}\right) \end{array}$$

(5) $$\begin{array}{cc} \; & p\left(a_{i}|x_{i}\right)=0\;\mathrm{or}\;1\;\forall \;i,a_{i},x_{i}\;(\mathrm{deterministic}). \end{array}$$

Equation (5) implies that for a single party with *m* measurement settings (inputs) and *d* possible outcomes (outputs), there are $d^{m}$ LDSs. For the m=2, d=3 case we consider in this work, the nine single-party LDSs are illustrated in Table 1.

From the single-party LDS and Equation (4), it is, in principle, possible to list all $d^{mN}$ vertices of P. Revealing nonlocality then consists of asking whether the observed statistics p(a|x) can be written as a convex combination of these vertices, which can be expressed as a linear program (LP) for which there exist efficient numerical algorithms. However, note that the exponential scaling in the number of parties makes such an approach unfeasible for large *N*.

Alternatively, it is possible to use computational geometry algorithms to convert the list of vertices (V-representation of P) into a list of inequality constraints (H-representation of P). These constraints specify the facets of the local polytope and thus correspond to tight Bell inequalities. Revealing nonlocality then consists of asking whether (at least) one of these inequalities is violated, as it would imply the impossibility of explaining the observed statistics by a LHVM.

In general, the number of inequalities can be much larger than the number of vertices (and, by duality, vice versa). A paradigmatic example where this occurs is the cross-polytope $C_{d}=\{x\in R^{d}{:\;||x||}_{1}\leq 1\}$, which has 2d vertices but $2^{d}$ inequalities. This worst-case exponential scaling manifests itself in dual-description algorithms that run in complexity as $O(v^{\left\lfloor D/2\right\rfloor })$, where *v* is the number of vertices, and *D* the polytope dimension [56].

Therefore, since the number of vertices scales exponentially with *N*, an exhaustive enumeration of all the vertices is typically impossible in a multipartite scenario. For example, in the (N,2,3) scenario considered here, N=10 already gives *v*∼$10^{9}$ vertices, which is already a prohibitive number for commonly available computing resources.

Deriving and characterizing the polytope of classical correlations for a multipartite system thus poses a formidable challenge. Already, for a few parties, the underlying combinatorial complexity [5,57] of the problem makes the task of listing all vertices or all facets unapproachable. For this reason, in order to partially overcome this complexity, it is often chosen to perform some simplification on the problem. This allows one to trade-off some nonlocality detection capability for the derivation of useful multipartite Bell inequalities. Moreover, we can do so in a way that the resulting inequalities fulfill desirable properties that make their experimental verification more approachable. An important strategy to simplify the complexity of the local polytope is discussed in the next section.

### 2.3. Projections onto Low-Dimensional Subspaces

**Figure 2:**
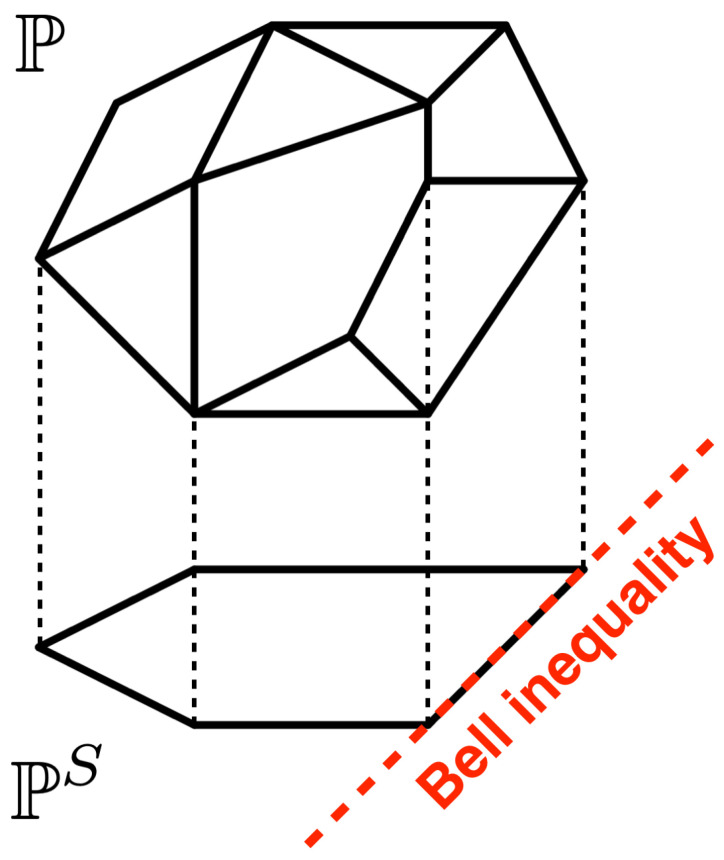
Illustration of the local polytope P and of its projection $P^{S}$ to a lower-dimensional subspace. The red dashed line indicates a resulting Bell inequality in the projected space.

A particularly successful approach to simplify the membership problem just mentioned consists of projecting P onto a lower-dimensional subspace. The resulting projection is also a polytope, $P^{S}$, of easier characterization because of the reduced dimensionality, see Figure 2. If the observed statistics p(a|x), after being appropriately projected, does not belong to $P^{S}$, then we must conclude that it also does not belong to P, and thus, that nonlocality is detected. On the contrary, if the projected p(a|x) lies inside $P^{S}$, then we cannot know whether p(a|x) lies inside P or not. For this reason, it is important to choose a projection that does not result in losing too much information about P, such that nonlocality can be detected, while still reducing the complexity of P to a computationally manageable level.

In the following, we consider a projection to the space of permutationally invariant (PI) one- and two-body conditional probabilities, which is defined by the observables

(6) $$\begin{array}{cc} P_{a|x} & :=\sum_{i}p\left(a_{i}|x_{i}\right)\;, \end{array}$$

(7) $$\begin{array}{cc} P_{ab|xy} & :=\sum_{i\neq j}p\left(a_{i}b_{j}|x_{i}y_{j}\right)\;. \end{array}$$

Limiting ourselves to one- and two-body marginals reduces the probability space from ${\left[m\left(d-1\right)+1\right]}^{N}-1$ dimensions (all possible p(a|x) minus the constraints in Equation (1)) to [Nm(d−1)+N2(m(d−1))2] dimensions. Despite this being a significant simplification, characterizing the projection of the local polytope in this subspace still requires enumerating all $d^{mN}=3^{2N}$ vertices. For this reason, we consider PI observables, which have two important consequences.

First, the dimension of the PI probability space defined by Equations (6) and (7) is reduced to m(d−1)+m(d−1)+m(d−1)2∈O(1) dimensions, which is independent of the number of parties *N*. Second, the vertices of the projected local polytope can now be parameterized much more efficiently: instead of having to consider all the $d^{mN}$ combinations specifying which party uses which LDS, permutational invariance implies that only knowing the number of particles using each LDS matters. Therefore, it is sufficient to consider partitions of *N* into $d^{m}$ integers, resulting in a list of points of polynomial size N+dm−1dm−1∼$O(N^{d^{m}-1}/\left(d^{m}-1\right)!)$. Some of these points will be vertices of the projected local polytope $P^{S}$, while the rest are in general interior points. The polynomial scaling may be improved to a lower degree by studying further the structure of the projected vertices. For instance, in [58], for the (N,2,2) scenario, the scaling could be improved from $N^{3}$ to $N^{2}$. We expect, but do not prove, that this improvement holds in more general scenarios. This is a consequence of most of the projected vertices not being extremal in the projection.

Keeping in mind the (N,2,3) scenario, let us denote with $c_{a,a^{\prime }}$ the total number of parties that have predetermined the pair of outcomes $a,a^{\prime }\in \left\{0,1,2\right\}$ for the two measurement settings x=0 and x=1, respectively. For example, $c_{2,0}$ is the number of parties giving as outcome a=2 when x=0 is measured and $a^{\prime }=0$ when x=1 is measured. That is, $c_{2,0}$ corresponds to the number of parties following the LDS #6 in Table 1. Note that $a,a^{\prime }$ corresponds to the expression of the LDS label in base *d* (so, $6=20_{\left(3\right)}$).

It follows by definition that the integers $c_{a,a^{\prime }}\geq 0$ and $\sum_{a,a^{\prime }}c_{a,a^{\prime }}=N$. Adopting this parametrization, it is possible to express the value of the PI one-body observables Equation (6) when computed on an LDS as

(8) $$\begin{array}{c} P_{a|x}\overset{LDS}{=}\left\{\begin{array}{cc} c_{a,0}+c_{a,1}+c_{a,2} & \mathrm{for}x=0\\ c_{0,a}+c_{1,a}+c_{2,a} & \mathrm{for}x=1 \end{array}\right.\;. \end{array}$$

Similarly, the PI two-body observables Equation (7) factorize under a given LDS as

(9) Pab|xy=LDS∑i≠jp(ai|xi)p(bj|yj)=∑i≠jp(ai|xi)p(bj|yj)+∑ip(ai|xi)p(bi|yi)⏟Pa|x·Pb|y==−∑ip(ai|xi)p(bi|yi)⏟:=Qab|xy,

where we have

(10) $$\begin{array}{c} Q_{ab,xy}=\left\{\begin{array}{c} P_{a|x}\\ 0\\ c_{a,b}\\ c_{b,a} \end{array}\right.\begin{array}{c} \;\mathrm{if}\;a=b,x=y\\ \;\mathrm{if}\;a\neq b,x=y\\ \;\mathrm{if}\;x=0,y=1\\ \;\mathrm{if}\;x=1,y=0 \end{array}\;. \end{array}$$

We summarize in Table 2 the one-body terms and the factorized two-body terms as a function of the quantities $c_{a,a^{\prime }}$. Note that, without loss of generality, we use the NS principle Equation (2) together with the normalization condition Equation (1) to neglect one of the outcomes (in this work, we choose to eliminate the (d−1)-th outcome). For example, we can write

p(02|xy)=1−p(00|xy)−p(01|xy)−p(10|xy)−p(11|xy)AA−p(12|xy)−p(20|xy)−p(21|xy)−p(22|xy)=1−p(00|xy)−p(01|xy)−p(1|x)−p(2|x)=1−p(00|xy)−p(01|xy)−(1−p(0|x))=p(0|x)−p(00|xy)−p(01|xy).

Hence, in our scenario, we can always consider Equations (8)–(10) with e.g., a,b∈{0,1} only. In addition, note that PI results in redundancies of the two-body observables Equation (7), such as $P_{01|10}=P_{10|01}$ or $P_{10|00}=P_{01|00}$. For this reason, for $P_{ab|xy}$, we take x≤y as canonical notation, and when x=y, we choose a≤b.

From the above considerations, we can conclude that the projected local polytope $P^{S}$ for the (N,2,3) scenario can be expressed in the 14-dimensional space of coordinates

(11) $$\begin{array}{c} \{P_{0|0},P_{1|0},P_{0|1},P_{1|1},P_{00|00},P_{01|00},P_{11|00},\\ \;P_{00|01},P_{01|01},P_{10|01},P_{11|01},P_{00|11},P_{01|11},P_{11|11}\}\;. \end{array}$$

The vertices of $P^{S}$ can thus be found by computing Equation (11) for all possible LDSs using the relations given in Table 2. This gives a list of N+32−132−1∼O(N8/8!) points, some of which are vertices. In the following, we are going to show how this list of points or, alternatively, the expressions in Table 2, can be used to derive useful three-outcome Bell inequalities for multipartite systems.

## 3. Deriving New Multipartite Bell Inequalities

After having characterized a projection of the local polytope in terms of its vertices, we are now interested in deriving Bell inequality capable of revealing Bell correlations in multipartite systems composed of three-level constituents. Since we consider a projection onto the space of permutationally invariant one- and two-body observables Equations (6) and (7), we call our inequalities 3-level PI Bell inequalities (3PIBIs). We start with presenting in Section 3.1 the more conventional (albeit somewhat involved) approach to derive new 3PIBIs, which consists of computing complete lists of inequalities for small *N* to then extrapolate how they generalize to arbitrary large *N*. Then, we present in Section 3.2 a data-driven method to derive a new inequality, which consists of using a numerical algorithm that approximates the local polytope from the outside to check membership for the experimentally observed statistics. The second method can be seen as checking all the inequalities defining an outer approximation to $P^{S}$ at once.

### 3.1. Inferring Families of 3-Outcome PIBIs

We have seen in Section 2.3 the approach of projecting the local polytope P for the (N,2,3) scenario onto a lower-dimensional subspace, which results in a polytope $P^{S}$ of much lower complexity: it lives in a space that is 14-dimensional, independently of the number of parties *N*, and its number of vertices scales as $N^{2}$.

For a given *N*, the vertices of $P^{S}$ are obtained by evaluating Equation (11) for all LDSs using Table 2. From this list of points, it is possible in principle to use computer algorithms [59] to obtain the dual description of $P^{S}$ in terms of intersecting half-spaces. Each one of these half-spaces is specified by an inequality constraint, which corresponds to a valid Bell inequality that is also tight (i.e., is a facet) for $P^{S}$. These take the form

(12) $$B=\sum_{a,x}\alpha_{a,x}P_{a|x}+\sum_{a,b,x,y}\alpha_{ab,xy}P_{ab|xy}+\beta_{c}\geq 0\;,$$

where the summations run over the terms in Equation (11), $\alpha_{a,x}\in R$, and the real number

(13) $$\beta_{c}:=-\min_{\mathrm{LDS}}\left(\sum_{a,x}\alpha_{a,x}P_{a|x}+\sum_{a,b,x,y}\alpha_{ab,xy}P_{ab|xy}\right)$$

is the so-called classical bound. In general, all the parameters α and $\beta_{c}$ depend on *N*. If a system exhibits statistics such that B<0, then it cannot be explained by an LHVM, and Bell nonlocality is detected.

In our analysis, we have been able to apply this procedure for N=2,3, which results in 165 and 146994 3PIBIs, respectively. It is interesting to note here that, while the number of vertices can be computed exactly to be $∼N^{8}$, the number of inequalities can have a severe scaling, in the worst case, $O(\mathrm{poly}{\left(N\right)}^{O(1)})$, which is still polynomial but of a worse degree. For N>3, the computational resources required by the algorithm converting vertices into facets is prohibitive, thus making it impossible to pursue this numerical approach further.

For this reason, in order to be able to find inequalities that are valid for a large number of parties, we impose an additional simplification to the problem. We decide to look only for 3PIBIs that are also invariant under the relabeling of inputs and outputs 0, 1. This choice can be motivated by physical arguments: one can imagine interactions in spin-1 systems, such as spin-exchanging collisions [52,53,54], that result in states that are invariant under the relabeling of $S_{z}=+1$ with $S_{z}=-1$. This additional symmetry we require for the 3PIBI can be enforced by introducing the following symmetrized one- and two-body observables

(14) $$\begin{array}{cc} {\tilde{P}}_{0} & :=P_{0|0}+P_{0|1}+P_{1|0}+P_{1|1} \end{array}$$

(15) $$\begin{array}{cc} {\tilde{P}}_{00} & :=P_{00|00}+P_{00|11}+P_{11|00}+P_{11|11} \end{array}$$

(16) $$\begin{array}{cc} {\tilde{P}}_{01} & :=P_{01|01}+P_{10|01} \end{array}$$

(17) $$\begin{array}{cc} {\tilde{P}}_{10} & :=P_{00|01}+P_{11|01} \end{array}$$

(18) $$\begin{array}{cc} {\tilde{P}}_{11} & :=P_{01|00}+P_{01|11}\;. \end{array}$$

The 14-dimensional space defined by Equation (11) of $P^{S}$ is thus projected further onto the 5-dimensional space with coordinates

(19) $$\{{\tilde{P}}_{0},{\tilde{P}}_{00},{\tilde{P}}_{01},{\tilde{P}}_{10},{\tilde{P}}_{11}\}\;.$$

Note that, by working in this subspace, we are effectively looking for 3PIBIs of the form

(20) $$B=\alpha_{1}{\tilde{P}}_{0}+\alpha_{2}{\tilde{P}}_{00}+\alpha_{3}{\tilde{P}}_{01}+\alpha_{4}{\tilde{P}}_{10}+\alpha_{5}{\tilde{P}}_{11}+\beta_{c}\geq 0\;,$$

which correspond to instances of Equation (12) with a very specific relation between the coefficients, namely $\alpha_{0,0}=\alpha_{0,1}=\alpha_{1,0}=\alpha_{1,1}$, $\alpha_{00,00}=\alpha_{00,11}=\alpha_{11,00}=\alpha_{11,11}$, $\alpha_{01,01}=\alpha_{10,01}$, $\alpha_{00,01}=\alpha_{11,01}$ and $\alpha_{01,00}=\alpha_{01,11}$.

With this additional projection, we are able to compute a full list of vertices and obtain from them complete lists of inequalities up to N=17, for which we find 1415 3PIBIs. Again, accessing even larger numbers of parties requires significant computational resources, thus becoming quickly unfeasible. However, having at hand complete lists of inequalities for 2≤N≤17 allows us to look for patterns in the coefficients, or for recurrent inequalities, and conjecture possible 3PIBIs that could be valid for arbitrarily large *N*. To give a concrete example, we propose for any N>3 the five 3PIBIs shown in Table 3.

At this point, for each conjectured inequality, we have to prove that it is indeed valid for an arbitrary number of parties *N*, or at least for all *N* larger than a minimum number. To this end, we use again the prescription given by Table 2 to write each inequality as a polynomial function in the coefficients $c_{a,a^{\prime }}$. Then, we want to prove that this function is non-negative when evaluated for integer values of the $c_{a,a^{\prime }}$. This ensures that the proposed 3PIBIs cannot be violated by an LHVM and that it is thus a valid Bell inequality. A concrete example of how this is carried out for PIBI #1 can be found in Refs. [60,61].

Finally, once a new Bell inequality is found, it is left to verify that it can be violated by appropriate measurements on a quantum state. This task is relatively easy to tackle in the (N,m,2) scenario [30,33,62], where one can consider *N* qubits on which Pauli measurements are performed among *m* directions (independent on the party). For the (N,2,3) scenario, the task of looking for a quantum violation can be significantly more tedious. In particular, parametrizing qutrit measurements from SU(3) operators is more involved. For this reason, we refer the reader interested in this search for a quantum violation to Refs. [60,61].

### 3.2. Data-Driven Derivation of 3-Outcome PIBIs

The approach to derive multipartite 3PIBIs proposed in the previous section consisted of deriving complete lists of inequalities for small *N*, to then look for inequalities that could be generalized to an arbitrary large *N*. However, due to the complexity of the problem, we had to further restrict our search to 3PIBIs that are invariant under the relabeling of some inputs and outputs, see Equation (20), therefore losing the possibility to find more general inequalities of the form Equation (12).

Here, we propose a complementary approach based on a generalization of Ref. [63] for an arbitrary number of outcomes. The general idea consists of approximating $P^{S}$ from the outside by the convex hull of a semialgebraic set (i.e., of a set defined by polynomial equalities and inequalities) to then write a semidefinite program (SdP) whose infeasibility is sufficient to certify that the observed statistics p(a|x) lies outside $P^{S}$ and, thus, that it exhibits nonlocal correlations. Importantly, SdPs form a class of well-behaved convex optimization problems which can be efficiently solved by numerical routines [64]. In addition, the dual variables of an infeasible SdP [65,66] provide a Bell inequality which is violated by p(a|x). Without going deep into the mathematical details of the method, in the following, we want to describe how to write such a SdP.

Our first task is to derive an outer approximation of $P^{S}$ in terms of the convex hull of a semialgebraic set. To this end, we consider the coefficients $c_{a,a^{\prime }}$ appearing in Table 2 to be non-negative real numbers, instead of positive integers. Then, we invert the expressions in Table 2 to write the conditions $c_{a,a^{\prime }}\in R_{\geq 0}$ in terms of the $P_{a|x}$, as well as to write the algebraic constraints between them. These are

(21) $$\begin{array}{c} \left\{\begin{array}{c} P_{0|0}^{2}-P_{0|0}-P_{00|00}=0\\ P_{0|0}P_{1|0}-P_{01|00}=0\\ P_{1|0}^{2}-P_{1|0}-P_{11|00}=0\\ P_{0|1}^{2}-P_{0|1}-P_{00|11}=0\\ P_{1|1}P_{0|1}-P_{01|11}=0\\ P_{1|1}^{2}-P_{1|1}-P_{11|11}=0 \end{array}\right. \end{array}$$

together with

(22) $$\begin{array}{c} \left\{\begin{array}{c} c_{0,0}=P_{0|0}P_{0|1}-P_{00|01}\geq 0\\ c_{0,1}=P_{0|0}P_{1|1}-P_{01|01}\geq 0\\ c_{0,2}=P_{0|0}-c_{0,0}-c_{0,1}=P_{0|0}-P_{0|0}P_{0|1}+P_{00|01}-P_{0|0}P_{1|1}+P_{01|01}\geq 0\\ c_{1,0}=P_{1|0}P_{0|1}-P_{10|01}\geq 0\\ c_{1,1}=P_{1|0}P_{1|1}-P_{11|01}\geq 0\\ c_{1,2}=P_{1|0}-c_{1,0}-c_{1,1}=P_{1|0}-P_{1|0}P_{0|1}+P_{10|01}-P_{1|0}P_{1|1}+P_{11|01}\geq 0\\ c_{2,0}=P_{0|1}-c_{0,0}-c_{1,0}=P_{0|1}-P_{0|0}P_{0|1}+P_{00|01}-P_{1|0}P_{0|1}+P_{10|01}\geq 0\\ c_{2,1}=P_{1|1}-c_{0,1}-c_{1,1}=P_{1|1}-P_{0|0}P_{1|1}+P_{01|01}-P_{1|0}P_{1|1}+P_{11|01}\geq 0\\ c_{2,2}=n-\sum_{\forall (a,b)\setminus (2,2)}c_{a,b}=\\ \;=n-P_{0|0}-P_{1|0}-P_{0|1}-P_{1|1}+P_{0|0}\left(P_{0|1}+P_{1|1}\right)-P_{00|01}-P_{01|01}+P_{1|0}\left(P_{0|1}+P_{1|1}\right)-P_{10|01}-P_{11|01}\geq 0 \end{array}\right. \end{array}$$

Note that Equation (22) is a set of nine inequality constraints ${\left\{g_{i}\left({\vec{P}}_{a|x}\right)\geq 0\right\}}_{i=1}^{9}$, and Equation (21) is a set of six equality constraints ${\left\{f_{i}\left({\vec{P}}_{a|x}\right)=0\right\}}_{i=1}^{6}$, which altogether define a semialgebraic set V approximating $P^{S}$ from the outside. This is a valid relaxation because, when $c_{a,a^{\prime }}$ are all evaluated at an actual integer partition of *n*, the P polytope coordinates correspond to a projected vertex of P.

Our task is now to certify that the observed statistics data point lies outside the set V, which, in turn, would imply that it is also outside of the local polytope $P^{S}$ and, thus, nonlocal. Deciding membership in the convex hull of a (semi)algebraic set is a subject of intensive research, but in its full generality, it is an NP-hard problem [67]. There exist, however, relaxations to this problem based on hierarchies of the SdP [63,68,69], which can be efficiently solved using numerical algorithms.

In this manuscript, we employ this relaxation approach, focusing specifically on the first level of the SdP hierarchy. As we will see, this already offers a robust outer approximation of the set of classical correlations, which is accurate enough for practical purposes. Even better approximations can be obtained by considering higher levels of the SdP hierarchy, at the expense of increasing computational costs. The first level consists of taking the union of {1} with the list in Equation (11) to obtain a basis set u as a column vector, which allows for constructing ten 15×15-dimensional moment matrices $\Gamma_{i}:=g_{i}\;u\;u^{T}$ for i∈{0,…,9}, with $g_{0}=1$ and $g_{i>0}$ as the expressions in Equation (22). For illustrative purposes, we write the matrix $\Gamma_{i}=\Gamma_{i}^{T}$ explicitly in Equation (23).

(23) $$\Gamma_{i}=g_{i}\left(\begin{array}{ccccccccccccccc} 1 & P_{0|0} & P_{1|0} & P_{0|1} & P_{1|1} & P_{00|00} & P_{01|00} & P_{11|00} & P_{00|01} & P_{01|01} & P_{10|01} & P_{11|01} & P_{00|11} & P_{01|11} & P_{11|11}\\ \cdot & P_{0|0}^{2} & P_{0|0}P_{1|0} & P_{0|0}P_{0|1} & P_{0|0}P_{1|1} & P_{0|0}P_{00|00} & P_{0|0}P_{01|00} & P_{0|0}P_{11|00} & P_{0|0}P_{00|01} & P_{0|0}P_{01|01} & P_{0|0}P_{10|01} & P_{0|0}P_{11|01} & P_{0|0}P_{00|11} & P_{0|0}P_{01|11} & P_{0|0}P_{11|11}\\ \cdot & \cdot & P_{1|0}^{2} & P_{1|0}P_{0|1} & P_{1|0}P_{1|1} & P_{1|0}P_{00|00} & P_{1|0}P_{01|00} & P_{1|0}P_{11|00} & P_{1|0}P_{00|01} & P_{1|0}P_{01|01} & P_{1|0}P_{10|01} & P_{1|0}P_{11|01} & P_{1|0}P_{00|11} & P_{1|0}P_{01|11} & P_{1|0}P_{11|11}\\ \cdot & \cdot & \cdot & P_{0|1}^{2} & P_{0|1}P_{1|1} & P_{0|1}P_{00|00} & P_{0|1}P_{01|00} & P_{0|1}P_{11|00} & P_{0|1}P_{00|01} & P_{0|1}P_{01|01} & P_{0|1}P_{10|01} & P_{0|1}P_{11|01} & P_{0|1}P_{00|11} & P_{0|1}P_{01|11} & P_{0|1}P_{11|11}\\ \cdot & \cdot & \cdot & \cdot & P_{1|1}^{2} & P_{1|1}P_{00|00} & P_{1|1}P_{01|00} & P_{1|1}P_{11|00} & P_{1|1}P_{00|01} & P_{1|1}P_{01|01} & P_{1|1}P_{10|01} & P_{1|1}P_{11|01} & P_{1|1}P_{00|11} & P_{1|1}P_{01|11} & P_{1|1}P_{11|11}\\ \cdot & \cdot & \cdot & \cdot & \cdot & P_{00|00}^{2} & P_{00|00}P_{01|00} & P_{00|00}P_{11|00} & P_{00|00}P_{00|01} & P_{00|00}P_{01|01} & P_{00|00}P_{10|01} & P_{00|00}P_{11|01} & P_{00|00}P_{00|11} & P_{00|00}P_{01|11} & P_{00|00}P_{11|11}\\ \cdot & \cdot & \cdot & \cdot & \cdot & \cdot & P_{01|00}^{2} & P_{01|00}P_{11|00} & P_{01|00}P_{00|01} & P_{01|00}P_{01|01} & P_{01|00}P_{10|01} & P_{01|00}P_{11|01} & P_{01|00}P_{00|11} & P_{01|00}P_{01|11} & P_{01|00}P_{11|11}\\ \cdot & \cdot & \cdot & \cdot & \cdot & \cdot & \cdot & P_{11|00}^{2} & P_{11|00}P_{00|01} & P_{11|00}P_{01|01} & P_{11|00}P_{10|01} & P_{11|00}P_{11|01} & P_{11|00}P_{00|11} & P_{11|00}P_{01|11} & P_{11|00}P_{11|11}\\ \cdot & \cdot & \cdot & \cdot & \cdot & \cdot & \cdot & \cdot & P_{00|01}^{2} & P_{00|01}P_{01|01} & P_{00|01}P_{10|01} & P_{00|01}P_{11|01} & P_{00|01}P_{00|11} & P_{00|01}P_{01|11} & P_{00|01}P_{11|11}\\ \cdot & \cdot & \cdot & \cdot & \cdot & \cdot & \cdot & \cdot & \cdot & P_{01|01}^{2} & P_{01|01}P_{10|01} & P_{01|01}P_{11|01} & P_{01|01}P_{00|11} & P_{01|01}P_{01|11} & P_{01|01}P_{11|11}\\ \cdot & \cdot & \cdot & \cdot & \cdot & \cdot & \cdot & \cdot & \cdot & \cdot & P_{10|01}^{2} & P_{10|01}P_{11|01} & P_{10|01}P_{00|11} & P_{10|01}P_{01|11} & P_{10|01}P_{11|11}\\ \cdot & \cdot & \cdot & \cdot & \cdot & \cdot & \cdot & \cdot & \cdot & \cdot & \cdot & P_{11|01}^{2} & P_{11|01}P_{00|11} & P_{11|01}P_{01|11} & P_{11|01}P_{11|11}\\ \cdot & \cdot & \cdot & \cdot & \cdot & \cdot & \cdot & \cdot & \cdot & \cdot & \cdot & \cdot & P_{00|11}^{2} & P_{00|11}P_{01|11} & P_{00|11}P_{11|11}\\ \cdot & \cdot & \cdot & \cdot & \cdot & \cdot & \cdot & \cdot & \cdot & \cdot & \cdot & \cdot & \cdot & P_{01|11}^{2} & P_{01|11}P_{11|11}\\ \cdot & \cdot & \cdot & \cdot & \cdot & \cdot & \cdot & \cdot & \cdot & \cdot & \cdot & \cdot & \cdot & \cdot & P_{11|11}^{2} \end{array}\right).$$

Next, in order to enforce our desired constraints, we apply the substitution rules given in Equation (21) to the entries of each $\Gamma_{i}$ and combine the resulting matrices to form a 150×150 block-diagonal matrix $\tilde{\Gamma }$. Finally, we linearize $\tilde{\Gamma }$ as $\tilde{\Gamma }\left(y\right)=\sum_{j}y_{j}{\hat{\Gamma }}_{j}$, where, for our case, $y=\{y_{j}\}$ with j∈{1,…,616} is a list of 616 real variables, and ${\hat{\Gamma }}_{j}$ are the associated 150×150 constant real matrices.

The problem of deciding whether the observed statistics $p={\left\{p\left(a|x\right)\right\}}_{a,x}$ lies outside $P^{S}$ can now be expressed as a feasibility problem via the SdP [63]

(24) $$\begin{array}{cc} \mathrm{find} & y\\ \mathrm{subject}\;\mathrm{to}\; & \tilde{\Gamma }\left(y\right)⪰0,\\ \; & y_{0}=1,\\ \; & y_{j}=\;p_{j}\;\forall \;j\in \left\{1,\ldots ,\dim\left(p\right)\right\}\;, \end{array}$$

where p is the obtained statistical data (c.f., Equation (11)), and y is the vector of decision variables (in our case, dim(y)=616). Note that in the decision variables, the components $y_{j}$ for j∈{0,…,dim(p)} are constrained, while the remaining $y_{j>\dim(p)}$ are left as free variables aiming to find a linear combination of ${\hat{\Gamma }}_{j}$ that makes $\tilde{\Gamma }\left(y\right)$ a positive semidefinite matrix.

If the SdP Equation (24) is feasible, it certifies that the point p lies within the outer approximation V. However, in this case, one cannot determine whether the point is local or nonlocal, as it could be within $P^{S}$ or in the gap between $P^{S}$ and V. Higher orders in the hierarchy of approximations might be considered in order to obtain a tighter approximation of $P^{S}$ by V and gain more insight into whether the point is local or nonlocal.

Conversely, if the SdP Equation (24) is infeasible, then it certifies that the point p lies outside the outer approximation V and, thus, outside $P^{S}$, signaling nonlocality. Importantly, when infeasible, one can obtain an analytical certificate of infeasibility in the form of a separating hyperplane (which exists by virtue of the Hahn–Banach Theorem [70]. In particular, if we name $\alpha =(\alpha_{1},\ldots ,\alpha_{\dim(p)})$ the dual variables associated with the constrained $y_{1},\ldots ,y_{\dim(p)}$ and $\beta_{c}$ the dual variable associated with $y_{0}$, then they define a Bell inequality in the form of Equation (12) that is violated by p. Namely, $\alpha \cdot p^{T}+\beta_{c}<0$ is a certificate that p is nonlocal. For more details on using the dual formulation of the SdP to provide certificates of infeasibility in the form of Bell inequalities, see, e.g., Section 3.1.5 of [65].

Finally, let us comment on some of the limitations of using this method. The first one we highlight is the lack of optimization methods in order to identify the suitable measurement settings to display nonlocality. That is, one must have an educated guess or implement the SdP for each different measurement setting. The second limitation we highlight is when one wants to have tighter approximations of the set. This can be achieved by going to higher levels in the hierarchy. However, note that advancing to a higher level in the hierarchy results in a significant increase in computational demands, and memory can become a limiting factor. In particular, the second level in the hierarchy is obtained by constructing the basis vector $u_{2\mathrm{nd}\text{-}\mathrm{lvl}}$ out of the elements appearing in the upper triangle of the matrix $uu^{T}$ (c.f. Equation (23)). This corresponds to a vector with 120 elements, leading to ten $\Gamma_{i}$ matrices of size 120×120, which makes the linearization process for obtaining the constraints more computationally expensive. Nonetheless, alternatively, if memory is a limitation but tighter approximations are desired, one can consider a pseudo-second level in the hierarchy by constructing the moment matrices from all first-order terms and a selection of second-order terms.

#### Benchmarking the Outer Approximation V against $P^{S}$

SdP Equation (24) can be adapted to provide a visualization of V and, for small values of *N*, compare it with a visualization of $P^{S}$. This allows us to benchmark the accuracy of our relaxations. We achieve this by posing the membership of p in V as the following optimization problem:

(25) $$\begin{array}{cccc} \max_{y,\lambda } & \lambda & \; & \\ \mathrm{subject}\;\mathrm{to}\; & \tilde{\Gamma }\left(y\right) & ⪰ & 0,\\ \; & \lambda & \geq & 0,\\ \; & y_{0} & = & 1,\\ \; & y_{j} & = & \lambda \;p_{j}\;\forall \;j\in \left\{1,\ldots ,\dim\left(p\right)\right\}\;, \end{array}$$

where the decision variables $y_{j}$ follow the same principle as in Equation (24), with the difference that, this time, we introduce the variable λ≥0, which scales the constraints associated with $y_{j}$ for j∈{0,…,dim(p)} to be proportional to the coordinates of p. This time, if the SdP returns a value λ≥1, then p lies within the relaxed set V, which does not tell us whether it is local or nonlocal. Conversely, if the SdP returns a value λ<1, then p lies outside of V, which certifies nonlocality. Moreover, when λ<1, its dual variables provide an analytical certificate of nonlocality in the form of a violated Bell inequality.

Next, in order to be able, to some extent, to visualize such high-dimensional sets, we proceed by selecting a two-dimensional plane in some (arbitrary) direction in order to obtain its intersection with the local polytope $P^{S}$ and its outer approximation V. Concretely, one can obtain the 2D slice of V with the following steps:

1. Select two (random) orthonormal directions $v_{1},v_{2}$ in the 14-dimensional space (cf. Equation (11)), defining the plane used to slice the local polytope.
2. Set as the origin the point $v_{\mathrm{mix}}$ inside the local polytope, which corresponds to the probability distribution of maximal entropy with $p(a_{i}|x_{i})=N/3$ and $p\left(a_{i}a_{j}|x_{i}x_{j}\right)=N\left(N-1\right)/9$ for all i,j.
3. Select a direction on the plane parametrized by an angle θ as $\tilde{v}=\cos\left(\theta \right)v_{1}+\sin\left(\theta \right)v_{2}$, noting that the discretization of θ need not be uniform to better outline the boundary.
4. Obtain the boundary points:
  - For V, find the max feasible λ along direction $\tilde{v}$ by inputting $p:=\tilde{v}+v_{\mathrm{mix}}/\lambda$ in SdP Equation (24). The $v_{\mathrm{mix}}/\lambda$ term is to obtain the constraint $y_{j}=\lambda {\tilde{v}}_{j}+{\left(v_{\mathrm{mix}}\right)}_{j},\;\forall \;j\in \left\{1,\cdots ,\dim\left(\tilde{v}\right)\right\}$. Then, one finds the boundary point $\lambda \tilde{v}$,
  - For $P^{S}$, find the max feasible μ such that $\mu \tilde{v}$ can be written as a linear combination of the vertices of $P^{S}$. We do so via the following linear program:
    (26) $$\begin{array}{cc} \max_{x} & (0,0,\ldots ,0,1)\cdot x\\ \mathrm{subject}\;\mathrm{to}\; & \left[A,{\left(0,\tilde{v}\right)}^{T}\right]x=v_{\mathrm{mix}},\\ \; & x_{i}\geq 0\;\forall i,\\ \; & x_{i}\leq 1\;\forall i, \end{array}$$
    where x is the decision variable with its last element corresponding to $x_{\dim(x)}\equiv \mu$, and $[A,{\left(0,\tilde{v}\right)}^{T}]x$ represents a matrix where the columns of *A* are the vertices of $P^{S}$ corresponding to all possible LDS configurations as outlined in Table 2. Then, one obtains the boundary point $\mu \tilde{v}$.
5. We repeat steps 3 and 4 for several values of θ∈{0,…,2π} until a full sweep across the plane has been completed.

**Figure 3:**
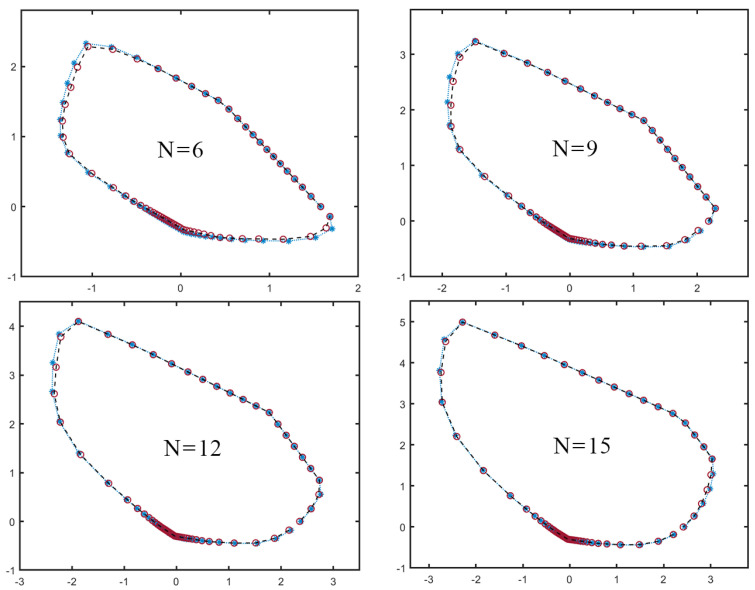
Slices of the local polytope $P^{S}$ (red circles and black dashed line), compared to a slice of its outer approximation provided by the SdP (26) (blue asterisks and blue dotted line), for different number of parties *N*. These slices are taken on a plane defined by two orthonormal directions $v_{1},v_{2}$ randomly chosen in the 14-dimensional space spanned by Equation (11). One observes that the outer approximation becomes relatively tighter as *N* increases [63].

We show in Figure 3 examples of slices taken in different planes and for different *N*. We see that, already for relatively small *N*, our SdP method tightly approximates the local polytope $P^{S}$. Crucially, this approximation is expected to improve even further as *N* increases [63].

## 4. Conclusions

We have discussed the problem of deriving new multipartite Bell inequalities in the (N,2,3) scenario, namely, when each of the *N* parties chooses to perform one out of two possible three-outcome measurements. As the complexity of fully characterizing the set of classical correlations becomes quickly intractable already for N>2, we consider a simplification to the problem: we focus on deriving Bell inequalities involving permutationally invariant one- and two-body observables. This is geometrically understood as characterizing only a projection of the full set of classical correlations onto a low-dimensional subspace. Importantly, imposing permutational invariance results in the dimension of this subspace being independent of the number of parties *N*.

We propose two complementary approaches for deriving three-outcome permutationally invariant Bell inequalities (3PIBIs). The first consists of deriving 3PIBIs for small N≤17, to then infer their possible generalization for an arbitrary large *N*. The second consists of approximating the projected set of classical correlations from the outside with a hierarchy of SdPs [63], which provides a certificate for non-membership of a point in the set taking the form of a valid 3PIBI.

The tools we propose have applications in deriving new Bell inequalities for the detection of nonlocality in paradigmatic three-level Hamiltonians [46,51], as well as for the construction of new experimentally friendly Bell correlation witnesses suited to ensembles of spin-1 particles [52,54]. These could enable the experimental detection of high-dimensional correlations in multipartite systems that are genuinely distinct from their two-level counterparts [61].

## Figures and Tables

**Table 1:** Local deterministic strategies p(a|x) in the 2-input 3-output scenario. Note that they satisfy $\sum_{a}p\left(a|x\right)=1$.

LDS Label	p(0|0)	p(1|0)	p(2|0)	p(0|1)	p(1|1)	p(2|1)
# 0	1	0	0	1	0	0
# 1	1	0	0	0	1	0
# 2	1	0	0	0	0	1
# 3	0	1	0	1	0	0
# 4	0	1	0	0	1	0
# 5	0	1	0	0	0	1
# 6	0	0	1	1	0	0
# 7	0	0	1	0	1	0
# 8	0	0	1	0	0	1

**Table 2:** Value of one- and two-body PI observables when computed for an LDS. The variables $c_{a,a^{\prime }}$ are non-negative integers satisfying $\sum_{a,a^{\prime }}c_{a,a^{\prime }}=N$, see main text.

$P_{0|0}\overset{LDS}{=}c_{0,0}+c_{0,1}+c_{0,2}$	$P_{00|00}\overset{LDS}{=}P_{0|0}^{2}-P_{0|0}$	$P_{00|01}\overset{LDS}{=}P_{0|0}P_{0|1}-c_{0,0}$	$P_{00|11}\overset{LDS}{=}P_{0|1}^{2}-P_{0|1}$
$P_{0|1}\overset{LDS}{=}c_{0,0}+c_{1,0}+c_{2,0}$	$P_{01|00}\overset{LDS}{=}P_{0|0}P_{1|0}$	$P_{01|01}\overset{LDS}{=}P_{0|0}P_{1|1}-c_{0,1}$	$P_{01|11}\overset{LDS}{=}P_{0|1}P_{1|1}$
$P_{1|0}\overset{LDS}{=}c_{1,0}+c_{1,1}+c_{1,2}$	$P_{11|00}\overset{LDS}{=}P_{1|0}^{2}-P_{1|0}$	$P_{10|01}\overset{LDS}{=}P_{1|0}P_{0|1}-c_{1,0}$	$P_{11|11}\overset{LDS}{=}P_{1|1}^{2}-P_{1|1}$
$P_{1|1}\overset{LDS}{=}c_{0,1}+c_{1,1}+c_{2,1}$		$P_{11|01}\overset{LDS}{=}P_{1|0}P_{1|1}-c_{1,1}$	

**Table 3:** Five proposed families of 3PIBIs, see Equation (20).

3PIBI Label	$\alpha_{1}$	$\alpha_{2}$	$\alpha_{3}$	$\alpha_{4}$	$\alpha_{5}$	$\beta_{c}$
# 1	1	1	0	−2	0	0
# 2	1	1	−2	−2	2	0
# 3	−2	1	2	2	0	4
# 4	−6	1	4	4	2	12
# 5	−6	1	4	0	0	24

## Data Availability

The code and data to construct the plots are available at the following link, https://github.com/Albert-Aloy/3PIBIs. Accessed on 23 September 2024.
